# Dynamic Recrystallization Constitutive Model and Texture Evolution of Metastable β Titanium Alloy TB8 during Thermal Deformation

**DOI:** 10.3390/ma17071572

**Published:** 2024-03-29

**Authors:** Chuankun Zhou, Fang Cao, Zhirong Yang, Weifeng Rao

**Affiliations:** 1 School of Mechanical Engineering, Qilu University of Technology (Shandong Academy of Sciences), Jinan 250353, China; 2 Shandong Institute of Mechanical Design and Research, Jinan 250353, China

**Keywords:** metastable β-titanium, TB8 titanium, hot-compression, microstructure evolution, dynamic recrystallization, processing map

## Abstract

The mechanical properties of metastable β-titanium alloys are highly susceptible during the thermal mechanical processing (TMP). In this process, the recrystallization process plays an important role in determining the microstructure and texture evolution. The implementation of dynamic recrystallization (DRX), a process for achieving β-grain refinement, is considered of great significance for the improvement of the properties of metastable β-titanium alloys and their industrial production. Along these lines, in this work, an isothermal compression test of TB8 titanium alloy was carried out by using a Gleeble-3500 thermal simulator. As a result, the rheological stress behavior was analyzed, the thermal processing map was accurately established based on the stress–strain curve, and the optimal processing interval was determined. The DRX kinetic and the DRX grain size models were developed, on the basis of which a new DRX intrinsic model was established to improve the material parameters. Therefore, the actual situation in the working process could be better predicted. The microstructural evolution of TB8 titanium alloy during thermal deformation was comprehensively investigated using the electron backscatter diffraction (EBSD) technique. The obtained results demonstrate a close correlation between the diversity of DRX mechanisms in TB8 alloy and the distribution of dislocation density. Four microstructural textures during thermal deformation were identified, in which the cube texture of (001) <010> and the R-Gorss Nd texture of (110) <110> dominate. Due to the random orientation of the dynamically recrystallized grains, the strength of the R-Gorss Nd texture of (110) <110> increases with the increase in the volume fraction of DRX. On the contrary, it was verified that the dynamic recrystallization behavior has a significant weakening impact on the cube texture of (001) <010>.

## 1. Introduction

Due to the excellent combination of mechanical properties, metastable β titanium alloys have gained more attention in the fields of aerospace, automotive parts, biomedicine, et al. [1,2,3]. It is now common to use TMP on titanium-alloy-microstructure-part modulation to obtain excellent mechanical properties, so that the parts have high creep properties and fracture toughness; these properties are very important for the manufacture of structural components. The mechanical properties of metastable β titanium alloys, however, exhibit high sensitivity to thermal mechanical processing (TMP), leading to deformation and hardening phenomena that significantly impact their mechanical performance and workability characteristics [4,5,6]. The literature has reported that beta grain refinement can enhance the ductility of alloys while concurrently augmenting their strength [7]. In other words, the process of dynamic recrystallization (DRX) can be utilized for β-grain refinement, resulting in small-grained alloys with high strength. Furthermore, titanium alloy DRX can also improve the macroscopic morphology of the material and enhance its plasticity and toughness. Therefore, it is of great significance to study the relationship between process parameters and microstructure as well as the texture evolution of metallic materials during thermal deformation.

For high-stacking-fault-energy titanium alloys, although thermal deformation does not readily induce dynamic recrystallization, numerous publications have highlighted that the primary recovery mechanism in the β-phase field is dynamic recovery (DRV), exhibiting an apparent activation energy similar to Ti diffusion in β-titanium alloys [8]. DRX is often observed during deformation above the β-phase transition temperature (T_β_) [9]. Continuous dynamic recrystallization (CDRX)and discontinuous dynamic recrystallization (DDRX) are the two primary types of DRX [10,11,12]. Arunabha et al. [13] studied Ti-6Al-4V alloy and found that DRX occurred during thermal deformation. During the initial deformation, DDRX dominated the nucleation mechanism of DRX, while CDRX gradually dominated DRX nucleation with the increase in true strain. Previous publications [14,15] have also confirmed that low strain rates and high deformation temperatures are conducive to DDRX, while CDRX readily occurs at high strain rates. Ti-6554 was studied using electron backscatter diffraction (EBSD) and it was discovered that CDRX prevailed at low strain rates. As the strain rate increased, the inhomogeneity of deformation caused multiple dynamic recrystallization mechanisms to occur simultaneously. DDRX was often found near jagged grain boundaries and rare geometric dynamic recrystallization (GDRX) was also detected at high strain rates [16]. Zhao et al. [17], during the compression of the Ti-10 V-2Fe-3Al alloy, found that DRV was the main recovery mechanism and that CDRX, which was characterized by continuous sub-grain rotation, readily occurred at low temperatures and high strain rates. However, there is a scarcity of research on the microstructure evolution of titanium alloys in relation to DRX mechanisms.

The constitutive equation Is used as an input to the user subroutine code of finite element software to simulate the deformation behavior of materials under certain conditions. It is very important in finite element simulation analysis and the optimization of material-hot-forming processing [18]. Therefore, the accuracy of finite element simulation heavily relies on the analysis of material deformation behavior through constitutive equations, which currently represent mathematical relationships between material flow behavior and parameters such as stress, strain, temperature, and strain rate. The majority of these constitutive equations are either phenomenological or empirical in nature [19]. In the phenomenological approach proposed by Sellars [20], the flow stress is described by the sinusoidal hyperbolic law within the Arrhenius equation, which has gained extensive utilization in predicting high-temperature flow behavior [16,21]. In the flow behavior modeling of titanium alloys, Teng et al. [22] employed the Arrhenius equation to simulate the flow behavior of the Ti-55511 alloy in both the two-phase and single-phase regions, successfully predicting the flow stress during thermal deformation with high accuracy. Furthermore, extensive research literature is available on the impact of various parameters on the flow behavior and microstructure evolution of Ti-55511 [23,24]. However, limited research has been conducted on the thermal deformation characteristics and DRX mechanism of TB8 alloys. Yang et al. [25] investigated the flow behavior and microstructure of TB8 alloy under hot compression at a strain rate of 0.001 s^−1^ and found that DRX occurred during the process. However, the thermal deformation behavior of TB8 prior to and subsequent to the phase transition has not been comprehensively investigated. Currently, superplastic forming and traditional thermal forming are the primary manufacturing methods employed for the production of intricate titanium alloy parts. However, these shaping techniques not only entail significant time and energy consumption but also incur exorbitant costs. [26]. Therefore, it is imperative to conduct a comprehensive analysis and investigation into the thermal deformation behavior, dynamic recrystallization (DRX) mechanism, and microstructural evolution of TB8 titanium alloy under varying strain rates, deformation temperatures, and levels of deformation. The DRX constitutive equation was developed to accurately predict the microstructure evolution and thermal deformation flow stress of TB8 titanium alloy, aiming to comprehensively comprehend the DRX mechanism during TB8 thermal deformation. The determination of the appropriate processing window for material hot forming, enhancement of material processing conditions, and improvement in forming efficiency through novel technologies are imperative for practical production.

In this work, the dynamic recrystallization and microstructure of TB8 titanium alloy were comprehensively analyzed at different temperatures, deformations, and strain rates. A new DRX-based intrinsic model was established based on the stress–strain curve, and a novel DRX kinetic model was developed. The microstructure evolution of the TB8 alloy during heat deformation was also systematically investigated. Our work provides valuable insights for studying the reasonable TMP process route to improve the properties of metastable β titanium alloy for industrialized production. The research results can provide theoretical basis and data support for TB8-alloy hot processing.

## 2. Experimental

The metastable β-titanium alloy TB8 that was used in the present study was hot-forged and annealed at 800 °C. A 200 × 200 mm^2^ block billet with a composition of Ti-15.23 Mo-2.93 Al-2.58 Nb-0.22 Si was obtained and then processed into cylindrical specimens with a diameter of 8 mm and a height of 12 mm. The T_β_ of the alloy was measured at 815 ± 15 °C by metallography.

**Figure 1:**
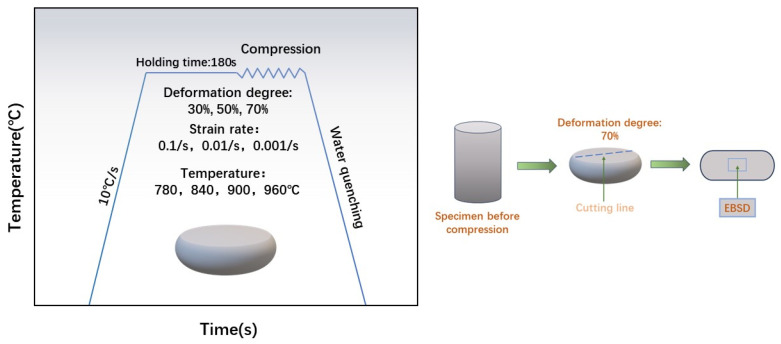
Thermal deformation flow chart and EBSD observation area diagram.

The hot compression experiments were performed on the cylindrical specimen using a Gleeble-3500 (The thermal simulation testing machine is developed by DSI company in Nashville, TN, USA) thermal simulation machine. All surfaces of each sample were mechanically ground to reduce friction. Between the squeezed specimen and die, thin tantalum sheets were positioned to further decrease friction and maintain consistent deformation. The thermal compression specification is shown in Figure 1: The samples were heated to 780, 840, 900, and 960 °C at 10 °C/s, held at 180 °C, compressed at a strain rate of 0.1–0.001 s^−1^ with 70% of the total deformation, and compressed at a strain rate of 0.001 s^−1^ at 960 °C with 30% and 50% of the deformation. The high-temperature microstructure was preserved by subjecting the samples to rapid water quenching immediately after thermal deformation. During the process of deformation, the system automatically collected true stress, true strain, and deformation temperature data. All compression tests were performed in a vacuum. The microstructural and textural evolution of the deformed specimens were analyzed by EBSD. Specimens after hot compression were cut along the central axis and subsequently ground using 400 #, 800 #, 1200 #, 2000 #, and 3000 # SiC sandpaper to achieve a smooth surface. Finally, mechanical polishing was performed utilizing 1 μm diamond polishing liquid. EBSD samples were prepared by grinding and electropolishing at 18 V for 15 s in 70 vol% CH_3_OH, 10 vol% HClO_4_, and 20 vol% (CH_2_OH)_2_ solutions. The observation area is shown in Figure 1. EBSD examination was carried out in ZEISS Sigma 500 (Jena, Germany) field-emission scanning electron microscope equipped with an Oxford Nordlys Max3 EBSD probe. The step size was 1.5 µm and the acceleration voltage was 20 kV. AZtecCrysta software (https://nano.oxinst.com/azteccrystal, accessed on 25 March 2024) was used to process and analyze EBSD data, and the DRX volume fraction, average grain size, average KAM and average GND density were obtained.

## 3. Results

### 3.1. Flow Behavior

**Figure 2:**
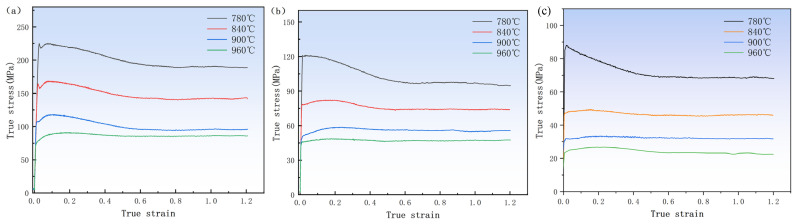
True stress–true strain curve of TB8 titanium alloy at high-temperature compression: (**a**) strain rate 0.1 s^−1^; (**b**) strain rate 0.01 s^−1^; (**c**) strain rate 0.001 s^−1^.

The curve in Figure 2 demonstrates a pronounced dependence of flow stress on the temperature and strain rate. Specifically, the flow stress increases with increasing strain rate at a given temperature. At a high strain rate, flow softening was reduced due to the rapid accumulation of dislocation, increasing critical shear stress. At the same strain rate, the flow stress decreased as the temperature increased and the flow stress became closer to the steady flow stress. This was because, at high temperatures, the binding force between atoms decreased, the critical shear stress of the material was reduced, the slip of the dislocation was accelerated, and the driving force of the dislocation slip decreased, thus reducing the work hardening. In addition, the stress–strain curves for various deformation cases have many common features. Early in the deformation process, the dislocation density rises rapidly and work hardening occurs. This caused the stress to rise quickly to its peak stress (σ_p_) across a very narrow strain range. The flow stress curve began a continuous softening stage following the peak stress as the strain continued to rise. It has been shown that the heat of deformation effect, DRV, DRX and rheological instability are all important components of the dynamic softening process [27]. The curve reached a steady state when work hardening and dynamic softening achieved equilibrium.

### 3.2. Hot Processing Maps

The hot processing map, based on the Dynamic Material Model (DMIM) proposed, serves as an effective tool for verifying thermal deformation mechanisms and optimizing thermal process parameters. It comprises a power dissipation diagram and instability diagram. The power dissipation diagram comprises the power dissipation coefficient (η), which exhibits variations with respect to deformation temperature and strain rate, and can be computed as follows:

(1) $$\eta ==\frac{J}{J_{\max}}=\frac{2m_{1}}{\left(m_{1}+1\right)}$$

In this context, J represents the power dissipation attributed to microstructural evolution, while m_1_ denotes the strain rate sensitivity index, which can be determined through the following equation:

(2) $$m_{1}=\frac{\partial (Ln\sigma )}{\partial (Ln\dot{\varepsilon })}{|}_{T}$$

By applying Zeigler’s principle of maximum entropy generation, the criterion for flow instability can be derived.

(3) $$\xi =\frac{\partial \left\{\mathrm{lg}\left[m_{1}/\left(m_{1}+1\right)\right]\right\}}{\partial \left(\mathrm{lg}\dot{\varepsilon }\right)}+m_{1}\;<\;0$$

**Figure 3:**
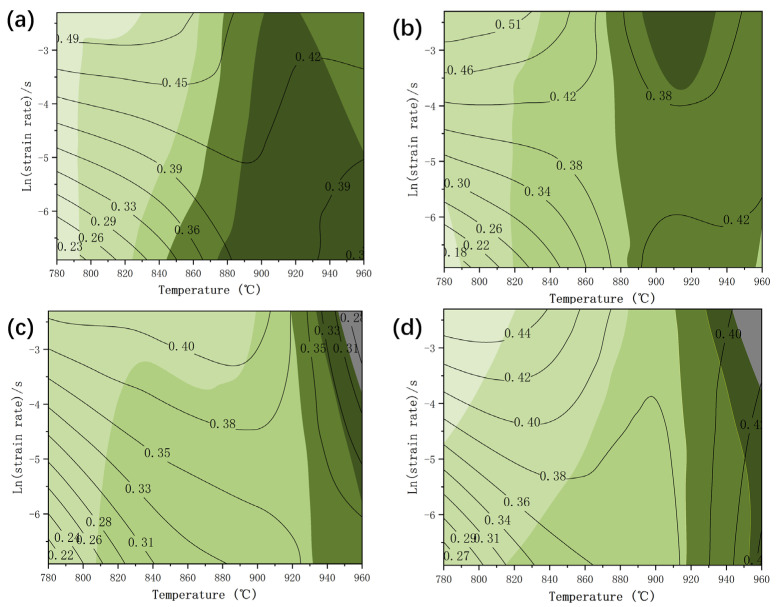
Hot processing map of TB8 titanium alloy under different true strains: (**a**) 0.3, (**b**) 0.6, (**c**) 0.9, and (**d**) 1.2.

Figure 3 depicts the hot processing map of the TB8 titanium alloy at different true stresses. The contours show the power dissipation efficiency; η ≥ 0.4 implies DRX or superplasticity, which can lower the deformation resistance and increase alloy workability [28]. Green denotes the safe zone, with the depth of the color representing stability; the darker color indicates poorer stability, while grey represents the unstable sector. The microstructure and performance can be optimized by choosing the processing parameters with the highest values and the region with the highest stability in the safe zone. As can be seen from Figure 3, the TB8 alloy had a high-power dissipation efficiency and a small instability zone. In Figure 3c,d, the gray color at a strain rate of 0.1 s^–1^ at 960 °C indicated instability. Thus, this region should be avoided as much as possible during hot working conditions. In contrast, η was greater than 0.38 and stable in the 780–900 °C region with a strain rate of 0.1–0.01 s^–1^, indicating that the workability was the best in this region.

**Figure 4:**
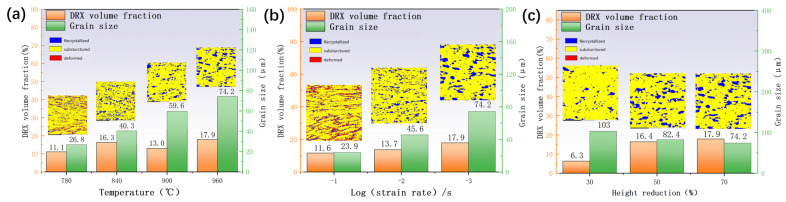
EBSD characterization results of TB8 titanium alloys under different deformation conditions: (**a**) strain rate 0.001 s^−1^, strain 1.2; (**b**) deformation temperature 960 °C, strain 1.2; (**c**) strain rate 0.001 s^−1^, temperature 960 °C.

Figure 4 illustrates a significant increase in average grain size with a rising temperature and decreasing strain rate, accompanied by the emergence of numerous deformed grains at a deformation temperature of 780 °C and a strain rate of 0.1 s^−1^. These grains are relatively large, affecting the alloy’s processing performance and mechanical properties. Combined with the thermal processing diagram in the strain rate 0.001 s^−1^ deformation temperature of 960 °C, it can be inferred that because the grain is larger, it will be easily destabilized in the processing. When the deformation temperature was 840–900 °C and the strain rate was 0.01 s^−1^ compared with the strain rate of 0.001 s^−1^ and the deformation temperature of 960 °C, the average grain size significantly decreased due to the dynamic recrystallization of β grains. It is thus apparent that the grain was refined, the alloy plasticity significantly increased, and the energy dissipation efficiency η increased. As can be seen from Figure 3, the energy dissipation efficiency η is greater than 0.38. Some regions even exceed 0.4 to appear superplastic. These results suggest that TB8 titanium alloy is best machined at a deformation temperature of 840–900 °C and a strain rate of 0.01 s^−1^.

### 3.3. Dynamic Recrystallization Constitutive Model

In this work, the correlation between deformation temperature T, flow stress σ, and strain rate ε during the compression deformation of TB8 titanium alloy is illustrated by using the Arrhenius equation [20]. The different stress levels were also divided into three types, as follows:

(4) $$\dot{\varepsilon }=A{\left[\sinh\left(\alpha \sigma_{p}\right)\right]}^{n}\exp\left(-Q_{a}/RT\right)$$

(5) $$\dot{\varepsilon }=A\sigma_{P}^{n}\exp\left(-Q_{a}/RT\right)$$

(6) $$\dot{\varepsilon }=A\exp\left(\beta \sigma_{P}\right)\exp\left(-Q_{a}/RT\right)$$

The parameters required for the DRX constitutive model can be obtained by fitting the above equation to the stress–strain curve [29].

The dynamic recrystallization constitutive model can be obtained as follows (see the Supplementary Materials):

(7) $$\left\{\begin{array}{l} \sigma_{0}=0.353511\cdot Z^{0.26082}\\ \sigma_{w}={\left[\sigma_{s}^{2}+\left(\sigma_{t}^{2}-\sigma_{s}^{2}\right)e^{-\mu \varepsilon }\right]}^{0.5}\cdots \cdots \left(\varepsilon_{q}<\varepsilon \leq \varepsilon_{c}\right)\\ \sigma_{d}=\sigma_{w}-\left(\sigma_{s}-\sigma_{ss}\right)X_{drx}\cdots \cdots \left(\varepsilon_{c}<\varepsilon \right)\\ Z=\dot{\varepsilon }\exp\left(239147/RT\right)\\ \varepsilon_{p}=1.730813\cdot Z^{-0.14929}\\ \varepsilon_{c}=0.83\varepsilon_{p}\\ \sigma_{s}=86.6446\times \sinh^{-1}(n_{s}Z^{d_{s}})\\ \sigma_{ss}=86.6446\times \sinh^{-1}(1.26\times 10^{-3}\cdot Z^{0.30876})\\ \mu =0.282995\cdot Z^{0.1341}\\ X_{drx}=1-\exp\left[K_{d}{\left(\left(\varepsilon -\varepsilon_{c}\right)/\varepsilon_{p}\right)}^{P_{d}}\right]\\ K_{d}=66.34577\cdot Z^{-0.19488}\\ P_{d}=3\times 10^{-4}\cdot Z^{0.31595}\\ D_{DRX}=1.45\times 10^{4}\cdot Z^{-0.3601}\\ d_{s}=0.01376\times \ln{\dot{\varepsilon }}^{2}+0.14417\ln\dot{\varepsilon }+0.60966\\ n_{s}=-0.14396\times 10^{-4}\times \ln{\dot{\varepsilon }}^{2}-0.00806\ln\dot{\varepsilon }-0.136 \end{array}\right.$$

In the equation, σ_t_ is the elastic stage stress, ε_q_ refers to the yield strain, σ_w_ is the instantaneous stress with only dynamic recovery, ε_c_ denotes the critical stress, εp is the peak strain, σ_d_ stands for the DRX stress, σ_s_ represents the peak stress, σ_ss_ signifies the steady-state stress, X_drx_ is the DRX volume fraction, and D_DRX_ states the DRX grain size.

**Figure 5:**
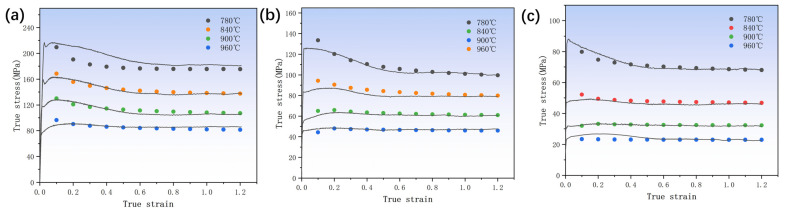
Comparison of predicted and experimental values of rheological stress at different strain rates: (**a**) 0.1 s^−1^; (**b**) 0.01 s^−1^; (**c**) 0.001 s^−1^.

The rheological stress values for thermal deformation at different strain rates and temperatures were calculated using Equation (7) and subsequently compared with the experimentally obtained stress–strain curves. The predictive ability of the new DRX intrinsic model of TB8 based on DRX dynamics and the dislocation density theory was tested. As shown in Figure 5, the prediction results are in good agreement with the experimental data.

## 4. Discussion

### 4.1. Dynamic Recrystallization Mechanism

**Figure 6:**
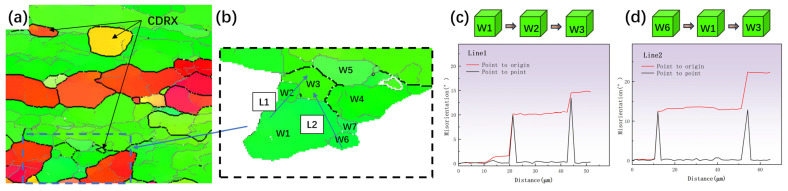
IPF diagram at 960 °C strain rate 0.001 s^−1^ deformation of 70 %: (**a**,**b**) partial enlarged drawing, and the distribution of the misorientation angle along (**c**) L1 and (**d**) L2.

**Figure 7:**
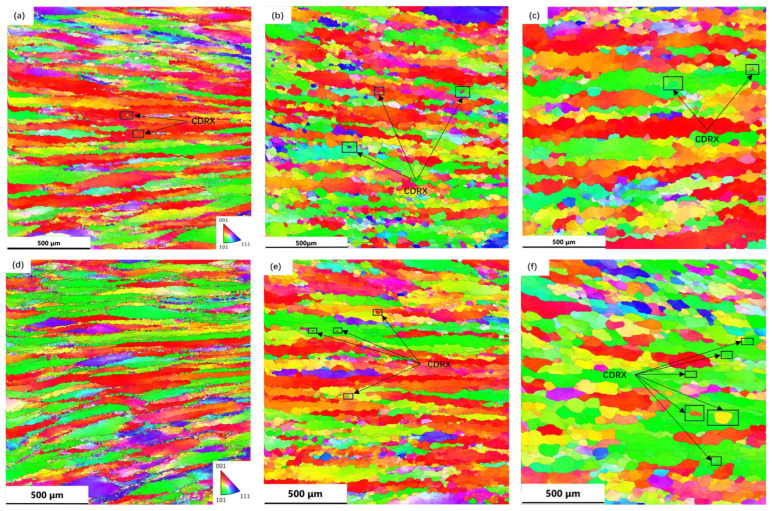
Inverse pole figures (IPFs) of TB8 titanium alloy at different temperatures and strain rates: (**a**) 780 °C, 0.001 s^−1^; (**b**) 840 °C, 0.001 s^−1^; (**c**) 900 °C, 0.001 s^−1^; (**d**) 960°C, 0.1 s^−1^; (**e**) 960 °C, 0.01 s^−1^; (**f**) 960 °C, 0.001 s^−1^.

Microstructure observations revealed that DRX occurred during the thermal deformation of the TB8 titanium alloy. The DRX phenomena mainly occurred in grain boundaries and grain interiors, suggesting that there were two main DRX mechanisms at play, namely, DDRX and CDRX. Similar phenomena have been also reported in other β-titanium alloys [16]. In Figure 6b, a local magnification of 70% deformation at 960 °C and a strain rate of 0.001 s^–1^ is shown. The figure shows that the original grain was segmented by low-angle grain boundaries (LAGBs) into many regular polygonal sub-grains (labelled W1–W7), which had the same color as the original grain. Additionally, the presence of sub-grain boundaries was evident in the comparison diagram. Figure 6c,d display the orientation-difference angle distribution of lines 1 and 2 along and within the original grain boundary, respectively. From Figure 6c, it can be observed that the cumulative orientation-difference distribution along line 1 presented three platforms and gradually increased, eventually approaching 15°. The point-to-point orientation difference at the sub-grain boundary suddenly changed, indicating that the sub-grain was relatively stable. The orientation of the W1–W3 sub-grains was similar but slightly rotated. Figure 6b also shows that part of the LAGB was transformed into high-angle grain boundaries (HAGBs). The above microstructural evolution indicated the presence of CDRX and the transition from LAGBs to HAGBs by the continual absorption of dislocations and the steady rotation of the lattice in Figure 6a, where the black arrows indicate the CDRX grains [30]. Zhang et al. [31] observed a similar phenomenon in Al-7.9Zn-2.7Mg-2.0Cu alloys. As shown in Figure 6d, the orientation-difference angle distribution of L2 exhibited a similar result, with an obvious orientation-difference gradient from the center to the edge of the sub-grains. The sub-grains gradually rotated and absorbed the dislocation, gradually changing from LAGBs to HAGBs and forming new DRX grains. This resulted in a differential orientation gradient from the center of the sub-grain to the edge [32]. As can be seen from Figure 7, CDRX gradually increased as the strain rate decreased. Combined with the results presented in Figure 7a–c, it can be observed that CDRX appeared at different temperatures when the deformation rate was 0.001 s^−1^. This effect indicates that the generation of CDRX is more dependent on the strain rate, and the nucleation of CDRX is more favorable at a low strain rate.

**Figure 8:**
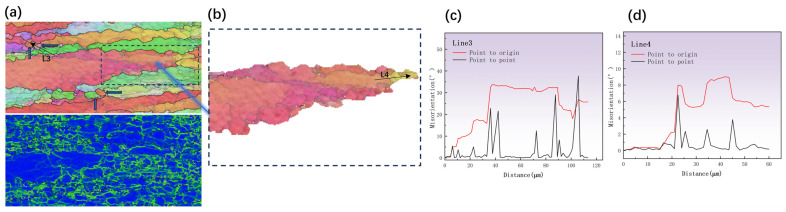
IPF and KAM diagrams at 780 °C, strain rate 0.001 s^–1^, deformation 70%: (**a**,**b**) partial enlargement, and the distribution of the misorientation angle along (**c**) L1 and (**d**) L2.

The blue arrow in Figure 8a indicates that there were some little DRX grains near the triple junction of the first grain. Similarly, some sub-grains were separated by sub-grain boundaries, which are typical of DDRX. It can be observed from Figure 8a’s enlarged IPF and kernel average misorientation (KAM) diagrams that the dislocation density inside the DBs was considerable, suggesting that the deformation energy storage there was relatively substantial and could easily trigger the DRX process [33]. As shown in Figure 8c, the distribution of orientation-difference angles along the L3 direction showed that the misorientation angles at the boundaries were all greater than 15°, indicating that the grains indicated by the blue arrow had developed into new DRX grains. The emergence of new DRX grains at the jagged boundary ridges of DBs is evidence of the occurrence of DDRX [34]. Figure 8d shows that the point-to-point orientation deviation in the sub-grains varied by less than 7° in the orientation-difference angle distribution diagram along the L4 direction. On average, the cumulative orientation error was much less than 15° and the bridging LAGB could also be observed in Figure 8b. According to earlier papers [33,35], the aforementioned characteristic is one of the prototypical features exhibited by DDRX. In general, the jagged grain boundaries in Figure 8 were caused by deformation coordination between the strain-induced boundary migration (SIBM) of the original HAGB and nearby grains. These sharp boundaries with significant local orientation gradients or fault density served as vantage locations for DDRX nucleation [36]. Due to the fact that DDRX is characterized by grain boundary growth, SIBM was crucial.

### 4.2. Effect of Strain Rate on Dislocation Distribution

**Figure 9:**
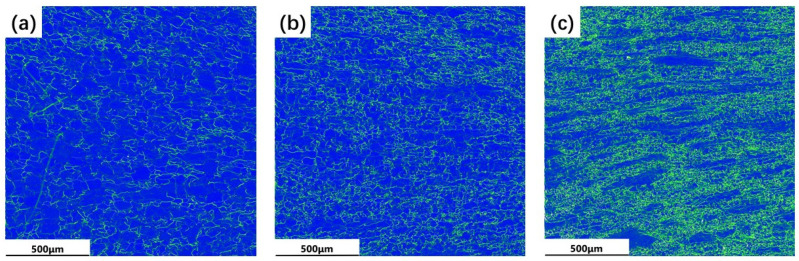
KAM maps of stacking grain boundaries of TB8 titanium alloy at different strain rates: (**a**) 0.001 s^−1^; (**b**) 0.01 s^−1^; (**c**) 0.1 s^−1^.

The strain rate affects the dislocation distribution [37]. Figure 9 shows the stacking grain boundary diagram of the KAM of the TB8 titanium alloy at different strain rates. Due to the increase in dislocation density and interaction, numerous dislocations aggregated and intertwined in local areas, forming an uneven distribution and resulting in the grains differentiating into many small crystals with slightly different dislocations. Therefore, the KAM chart could be used to characterize the non-uniform deformation of the material and observe its dislocation density distribution. The storage of deformation energy and dislocation density, which correlate to KAM in the figure, are both quite high. The microstructure’s dislocation density distribution, which is closely related to the energy difference that causes dislocations to develop between grains, dictates the circumstances under which DRX might arise [38]. Therefore, the KAM values can be employed to estimate the distribution of geometrically necessary dislocation (GND) density, enabling an analysis of variations in dislocation density during thermal deformation of titanium alloy under different strain rates, as follows [39]:

$$\rho_{G}=\frac{2K_{AVE}}{\mu B}$$

**Figure 10:**
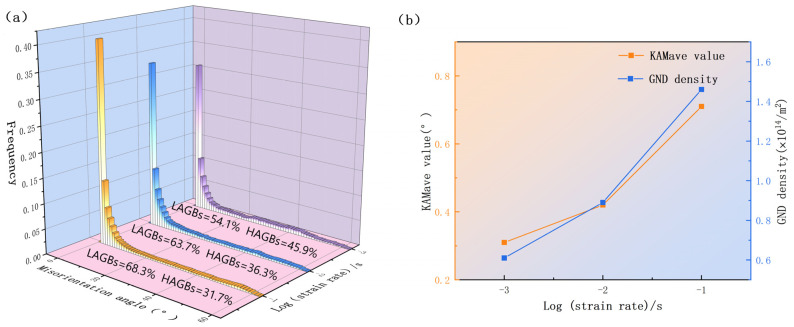
EBSD characterization results of TB8 titanium alloys at different strain rates: (**a**) orientation-difference distribution; (**b**) line plots of KAM mean and GND mean density statistics.

Here, ρ_G_ represents the GND density, K_AVE_ represents the mean KAM value, μ represents the step size of the EBSD test, B is the Burgers vector. As seen in Figure 9a, at low strain rates, the dislocation was mostly distributed along the grain borders, with low KAM within the grains and a very uniform distribution of dislocations near the grain boundaries. A comparison of the inverse level diagram and stacked grain boundary diagrams through Figure 9a reveals that the level of KAM values is higher near the LAGB than near the HAGB. KAM and GND densities on average are shown in Figure 10. When compared to other strain rates, the average KAM was 0.001 s^−1^ and the corresponding GND density was at its lowest. Li et al. [38] demonstrated in their investigation of Ti-6554 that the occurrence of DRX can effectively mitigate dislocation density and deformation energy during the deformation process.

Figure 9b illustrates that the dislocation density at a strain rate of 0.01 s^–1^ gradually moved towards the sawtooth grain boundary and inside the DBs. The existence of a higher strain rate was conducive to the proliferation of dislocation. The average KAM and GND densities increased to 0.42° and 0.89 × 10^14^ m^–2^, respectively (Figure 10), and numerous dislocations accumulated at the grain boundaries because there was insufficient time to develop DRX under such conditions. The average KAM and GND densities reached their maximum values of 0.71° and 1.46 × 10^14^ m^−2^, respectively, when the strain rate was raised further to 0.1 s^−1^. As mentioned above, very serious non-uniform deformation occurred inside the material at high strain rates. Under the action of strong localized stress concentrations, the grains elongated. This was due to the high dislocation density and substantial deformation energy storage within the grain boundary, which supported the growth of the DRX process. Some of the dislocation density was also used up during the DRX nucleation process. As shown in Figure 10, the number percentage of LAGB significantly decreased with the decrease in the strain rate, which also proves that LAGB absorbs dislocations and transforms into HAGB with the decrease in the strain rate. On the contrary, the growth rate of the KAM average and GND density at strain rates from 0.001 s^−1^ to 0.01 s^−1^ was smaller than that of strain rates from 0.01 s^−1^ to 0.1 s^−1^, which is also in line with the above-mentioned viewpoints and underlines the validity of the model.

### 4.3. Textural Evolution

The texture that arises during the hot working process has a considerable impact on the mechanical properties of the TB8 titanium alloy. Therefore, it is essential to describe in detail how the TB8 titanium alloy’s textural evolution occurred during the application of the thermomechanical machining process. The microscopic texture of a deformed material may be quantified by tracing the three-dimensional orientation distribution function (ODF), which can describe the texture intensity of each orientation in three dimensions. Each texture component in the ODF section is made up of the Euler angles (φ_1_, ϕ, φ_2_). The color’s depth reflects the texture’s intensity. By employing an ODF cross-section of φ_2_ = 45°, it becomes feasible to analyze the conventional texture composition of body-centered cubic (bcc) metallic materials [40].

**Figure 11:**
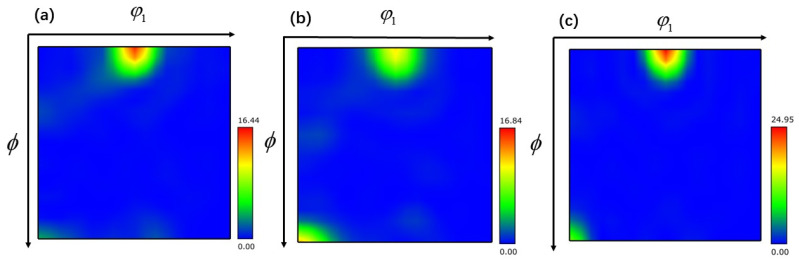
ODF cross-section of TB8 titanium alloy at different temperatures φ_2_ = 45°: (**a**) 780 °C; (**b**) 840 °C; (**c**) 900 °C.

**Figure 12:**
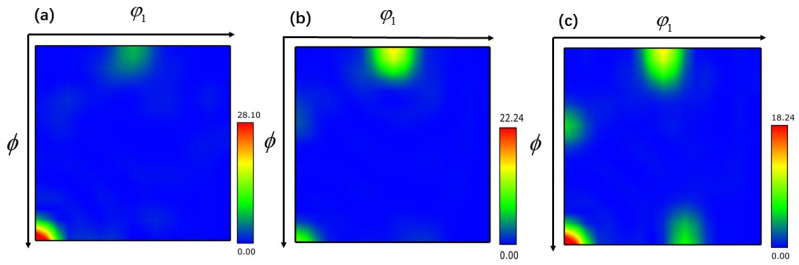
ODF cross-section of TB8 titanium alloy at different strain rates φ_2_ = 45°: (**a**) 0.001 s^−1^; (**b**) 0.01 s^−1^; (**c**) 0.1 s^−1^.

**Figure 13:**
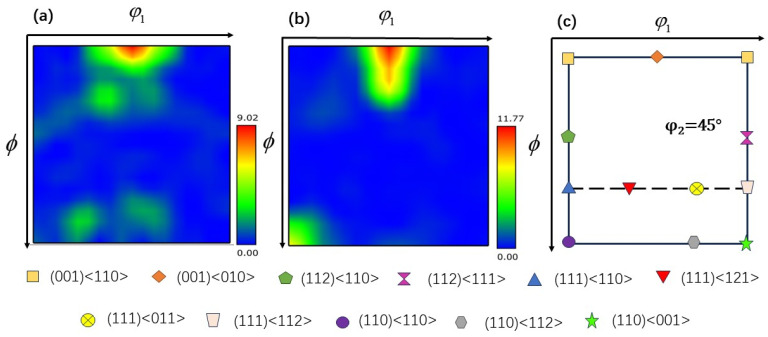
ODF cross-section of TB8 titanium alloy under different deformations of φ_2_ = 45°: (**a**) 30%; (**b**) 50%; (**c**) cross-section diagram of ODF.

The ODF cross-section of TB8 titanium alloy is illustrated in Figure 11, Figure 12 and Figure 13. The main components of the deformed samples at different temperatures were the same, comprising the following types: (001) <010> cube texture, (110) <110> R-Gorss Nd texture, (110) <110> R-Gorss Nd texture, and (110) <112> brass texture. In particular, the (001) <010> cube texture and (110) <110> R-Gorss Nd texture predominated. It is widely accepted that R-Gorss Nd texture, brass texture, and cube texture are the most relevant textural components for compressing BCC metal materials [41].

**Figure 14:**
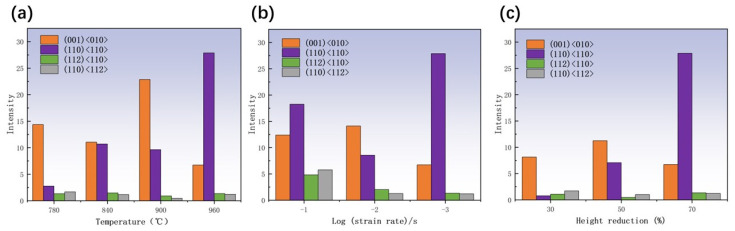
Statistical diagram of different texture strengths of TB8 titanium alloy: (**a**) different temperatures; (**b**) different strain rates; (**c**) different amounts of deformation.

Figure 14a presents the statistics of the textural strength of TB8 titanium alloy at different temperatures with a strain rate of 0.001 s^–1^ and 70% deformation. Compared with the results depicted in Figure 4a, it can be observed that the strength of the cube texture decreased as the DRX volume fraction increased. This result indicated that the generation of DRX reduced the strength of the cube texture. At 960 °C, the greatest generation of DRX occurred, and the strength of the cube texture was also the lowest. In contrast, the strength of the R-Gorss Nd texture increased as the DRX volume increased, confirming that the generation of DRX contributed to the strength of the R-Gorss Nd texture. Figure 14b shows the textural strength of the TB8 titanium alloy under different strain rates at 960 °C and 70% deformation. As can be seen from the figure, the cube texture decreased when the R-Gorss Nd texture increased, indicating that the appearance of R-Gorss Nd texture had an inhibitory effect on the cube texture. Furthermore, the strength of the cube texture and brass texture decreased at decreasing strain rates, in contrast to the DRX volume fraction that increased with the decreasing strain rate (Figure 4b). All these results indicated that the generation of DRX weakened the strength of the cube and brass textures.

**Figure 15:**
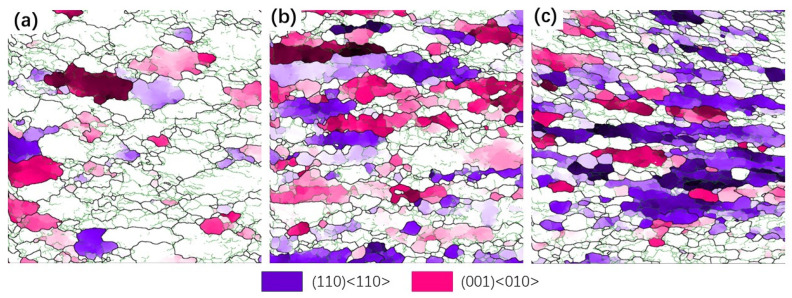
Nucleation position distribution of TB8 alloy by EBSD analysis under different deformation amounts (001) <010> and (110) <110> orientation texture. (**a**) 30%; (**b**) 50%; (**c**) 70%.

As shown in Figure 14c, as the deformation increased from 30% to 70%, the strength of the (110) <110> texture significantly increased, while the strength of the (001) <010> texture first increased and then decreased. This effect could be interpreted by taking into account the place where grains had recrystallized nucleated. As can be observed in Figure 15, the majority of the (110) <110> R-Gorss Nd texture was found in the recrystallized grains that had protruded and nucleated at the primary grain boundary. In striking contrast, a minor number was found in the recrystallized grains that had nucleated inside the primary grains and had undergone deformation at the three nodes of the primary grains. When metals are thermally deformed, the grain boundary expansion nucleation caused by SIBM plays a key role in the recrystallization process of deformed metals [12]. Figure 15 further demonstrates that the majority of the (001) <010> cube texture developed in the highly deformed original grains, with just a small quantity created in the recrystallized grains with the nucleation of the original grain borders. The volume percentage of recrystallized grains and the degree of deformation in the original grains exhibited a gradual increase as the deformation level reached 50%. A similar pattern was detected for the intensity of the associated (001) <010> cube texture. DRX was totally created, and the original grains were gradually absorbed when the deformation amount reached 70%. As a result, the (001) < 010> cube texture began to progressively decline.

## 5. Conclusions

The recrystallization mechanism and microstructure evolution of TB8 alloy during high-temperature deformation were thoroughly studied in this work. The microstructure evolution and DRX mechanism of the materials were analyzed, and the following conclusions can be drawn:

1. The strain rate exerts a greater influence on the flow properties of the stress–strain curve of TB8 alloy compared to the deformation temperature. Moreover, at low temperatures and high strain rates, the deformed alloy exhibits more pronounced rheological softening. DRX occurs in isothermal compression near the β-phase region, and the diversity of DRX mechanisms in TB8 alloys is closely associated with the distribution of the dislocation densities. The distribution of the high dislocation density is also associated with non-uniform deformation, and dynamic recrystallization (DRX) occurs in certain elongated grains and deformation bands (DBs) due to the impact of localized energy accumulation. This includes CDRX, which is characterized by the transition from LAGB to HAGB, and DDRX, and takes place by grain boundary expansion. DRX is strongly dependent on the employed process parameters. The low strain rate is more conducive to the nucleation of CDRX.
2. With the increase in the compression temperature and strain rate, the stability of TB8 titanium alloy deteriorates. The stability starts to get gradually better when the temperature exceeds 900 °C, and destabilization occurs when the compression temperature is at 960 °C and the strain rate is 0.001 s^−1^. The optimal processing interval can be found between 840 and 900 °C at a strain rate of 0.01 s^−1^.
3. The constitutive model of TB8 titanium alloy for dynamic recrystallization was established based on the stress–strain curve, employing the DRX dynamic model and dislocation density theory. In order to enhance the precision of flow stress prediction during dynamic recrystallization, refinements were made to the material parameters.
4. The (001) <010> cube texture, (110) <110> R-Gorss Nd texture, (112) <110> texture, and (110) <112> brass texture were found in the TB8 titanium alloy. The (001) <010> cube texture and (110) <110> R-Gorss Nd texture predominated. Due to the random orientation of DRX grains, the intensity of (110) <110> R-Gorss Nd texture increased as the DRX volume fraction increased and the DRX behavior of (001) <010> cube texture had a significant weakening effect.

## Data Availability

The data that support the findings of this study are available from the corresponding author upon reasonable request.

## Supplementary Materials

The following supporting information can be downloaded at: https://www.mdpi.com/article/10.3390/ma17071572/s1, this section mainly includes some unimportant information in the manuscript, including the DRX constitutive model building process and the inverse pole figure of TB8 titanium alloy, with the aim of increasing its completeness [29,42,43].

## References

[1] Awad A.H., Aly H.A., Saood M. (2024). Physical, mechanical, and corrosion properties of Ti-12Mo and Ti-15Mo alloys fabricated by elemental blend and mechanical alloying techniques. Mater. Chem. Phys..

[2] Hulka I., Florido-Suarez N.R., Mirza-Rosca J.C., Saceleanu A. (2022). Mechanical Properties and Corrosion Behavior of Thermally Treated Ti-6Al-7Nb Dental Alloy. Materials.

[3] Kozadaeva M., Surmeneva M., Khrapov D., Rybakov V., Surmenev R., Koptyug A., Vladescu A., Cotrut C.M., Tyurin A., Grubova I. (2023). Assessment of Microstructural, Mechanical and Electrochemical Properties of Ti-42Nb Alloy Manufactured by Electron Beam Melting. Materials.

[4] Kodzhaspirov G.E., Rudskoy A.I., Borowikow A. Thermomechanical processing of Ti and Nb-alloyed stainless steels. Presented at the International Conference Rolling.

[5] Premkumar M., Himabindu V.S., Banumathy S., Bhattacharjee A., Singh A.K. (2012). Effect of mode of deformation by rolling on texture evolution and yield locus anisotropy in a multifunctional β titanium alloy. Mater. Sci. Eng. A.

[6] Ahmed M., Wexler D., Casillas G., Ivasishin O.M., Pereloma E.V. (2015). The influence of β phase stability on deformation mode and compressive mechanical properties of Ti-10V-3Fe-3Al alloy. Acta Mater..

[7] Ouyang D.L., Fu M.W., Lu S.Q. (2014). Study on the dynamic recrystallization behavior of Ti-alloy Ti-10V-2Fe-3V in β processing via experiment and simulation. Mater. Sci. Eng. A Struct. Mater. Prop. Microstruct. Process..

[8] Jones N.G., Dashwood R.J., Dye D., Jackson M. (2008). Thermomechanical processing of Ti-5A1-5Mo-5V-3Cr. Mater. Sci. Eng. A Struct. Mater. Prop. Microstruct. Process..

[9] Chuan W., Liang H. (2018). Hot deformation and dynamic recrystallization of a near-beta titanium alloy in the beta single phase region. Vacuum.

[10] Warchomicka F., Poletti C., Stockinger M. (2011). Study of the hot deformation behaviour in Ti-5Al-5Mo-5V-3Cr-1Zr. Mater. Sci. Eng. A Struct. Mater. Prop. Microstruct. Process..

[11] Dikovits M., Poletti C., Warchomicka F. (2014). Deformation Mechanisms in the Near-β Titanium Alloy Ti-55531. Metall. Mater. Trans. A Phys. Metall. Mater. Sci..

[12] Hasegawa M., Yamamoto M., Fukutomi H. (2003). Formation mechanism of texture during dynamic recrystallization in γ-TiAl, nickel and copper examined by microstructure observation and grain boundary analysis based on local orientation measurements. Acta Mater..

[13] Roy A.M., Arróyave R., Sundararaghavan V. (2023). Incorporating dynamic recrystallization into a crystal plasticity model for high-temperature deformation of Ti-6Al-4V. Mater. Sci. Eng. A Struct. Mater. Prop. Microstruct. Process..

[14] Li C.M., Huang L., Zhao M.J., Guo S.Q., Li J.J. (2021). Hot deformation behavior and mechanism of a new metastable β titanium alloy Ti-6Cr-5Mo-5V-4Al in single phase region. Mater. Sci. Eng. A Struct. Mater. Prop. Microstruct. Process..

[15] Li L., Luo J., Yan J.J., Li M.Q. (2015). Dynamic globularization and restoration mechanism of Ti-5Al-2Sn-2Zr-4Mo-4Cr alloy during isothermal compression. J. Alloys Compd..

[16] Zhang D., Dong X.J., Xu Y., Lu S.Q., Wei K., Huang L. (2023). Dynamic recrystallization mechanism of Ti-6554 alloy during high-temperature deformation. J. Alloys Compd..

[17] Zhao J., Zhong J., Yan F., Chai F., Dargusch M. (2017). Deformation behaviour and mechanisms during hot compression at supertransus temperatures in Ti-10V-2Fe-3A1. J. Alloys Compd..

[18] Bontcheva N., Petzov G., Parashkevova L. (2006). Thermomechanical modelling of hot extrusion of Al-alloys, followed by cooling on the press. Comput. Mater. Sci..

[19] Haghdadi N., Zarei-Hanzaki A., Khalesian A.R., Abedi H.R. (2013). Artificial neural network modeling to predict the hot deformation behavior of an A356 aluminum alloy. Mater. Des..

[20] Sellars C.M., McTegart W.J. (1966). On the mechanism of hot deformation. Acta Metall..

[21] Shafaat M.A., Omidvar H., Fallah B. (2011). Prediction of hot compression flow curves of Ti-6Al-4V alloy in α plus β phase region. Mater. Des..

[22] Teng H.H., Xia Y.F., Sun T., Zheng D.Y., Chen L. (2023). Flow Stress Prediction of Near-β Ti-55511 Alloy During Isothermal Compression Based on Corrected Arrhenius Model with Material Parameter Evolution and BP-ANN Model. Rare Met. Mater. Eng..

[23] Shi S.X., Liu X.S., Zhang X.Y., Zhou K.C. (2021). Comparison of flow behaviors of near beta Ti-55511 alloy during hot compression based on SCA and BPANN models. Trans. Nonferrous Met. Soc. China.

[24] Kumar V.A., Murty S., Gupta R.K., Rao A.G., Prasad M. (2020). Effect of boron on microstructure evolution and hot tensile deformation behavior of Ti-5Al-5V-5Mo-1Cr-1Fe alloy. J. Alloys Compd..

[25] Yang Q.Y., Ma M., Tan Y.B.A., Xiang S., Zhao F., Liang Y.L. (2021). Microstructure and texture evolution of TB8 titanium alloys during hot compression. Rare Met..

[26] Guo M.L., Liu J., Tan M.J., Chua B.W. Microstructure evolution of Ti-6Al-4V during superplastic-like forming. Proceedings of the 11th International Conference on Technology of Plasticity (ICTP).

[27] Zhou L., Cui C., Wang Q.Z., Li C., Xiao B.L., Ma Z.Y. (2018). Constitutive equation and model validation for a 31 vol.% B4Cp/6061Al composite during hot compression. J. Mater. Sci. Technol..

[28] Lei J., Zhu W.G., Chen L., Sun Q.Y., Xiao L., Sun J. (2020). Deformation behaviour and microstructural evolution during the hot compression of Ti-5Al4Zr8Mo7V alloy. Mater. Today Commun..

[29] El Wahabi M., Cabrera J.M., Prado J.M. (2003). Hot working of two AISI 304 steels: A comparative study. Mater. Sci. Eng. A Struct. Mater. Prop. Microstruct. Process..

[30] Li J.C., Wu X.D., Cao L.F., Liao B., Wang Y.C., Liu Q. (2021). Hot deformation and dynamic recrystallization in Al-Mg-Si alloy. Mater. Charact..

[31] Zang Q.H., Yu H.S., Lee Y.S., Kim M.S., Kim H.W. (2019). Effects of initial microstructure on hot deformation behavior of Al-7.9Zn-2.7Mg-2.0Cu (wt%) alloy. Mater. Charact..

[32] Che B., Lu L.W., Wu Z.Q., Zhang H., Ma M., Luo J., Zhao H.M. (2021). Dynamic recrystallization behavior and microstructure evolution of a new Mg-6Zn-1Gd-1Er alloy with and without pre-aging treatment. Mater. Charact..

[33] Fan X.G., Zhang Y., Gao P.F., Lei Z.N., Zhan M. (2017). Deformation behavior and microstructure evolution during hot working of a coarse-grained Ti-5Al-5Mo-5V-3Cr-1Zr titanium alloy in beta phase field. Mater. Sci. Eng. A Struct. Mater. Prop. Microstruct. Process..

[34] Gupta A., Khatirkar R., Singh J. (2022). A review of microstructure and texture evolution during plastic deformation and heat treatment of beta-Ti alloys. J. Alloys Compd..

[35] Balasubrahmanyam V.V., Prasad Y. (2002). Deformation behaviour of beta titanium alloy Ti-10V-4.5Fe-1.5Al in hot upset forging. Mater. Sci. Eng. A Struct. Mater. Prop. Microstruct. Process..

[36] Sun Y.G., Zhang C.J., Feng H., Zhang S.Z., Han J.C., Zhang W.G., Zhao E.T., Wang H.W. (2020). Dynamic recrystallization mechanism and improved mechanical properties of a near alpha high temperature titanium alloy processed by severe plastic deformation. Mater. Charact..

[37] Li Q.K., Yan H., Liu H.H., Chen R.S. (2022). Dynamic recrystallization mechanism and near-isotropic mechanical properties of WE43 magnesium alloy sheets rolled at different temperatures. Mater. Charact..

[38] Li C.M., Huang L., Zhao M.J., Guo S.Q., Li J.J. (2022). Study on microstructure evolution and deformation mechanism of Ti-6554 based on power dissipation efficiency at supertransus temperatures. J. Alloys Compd..

[39] Xu Z.N., Xu L.J., Xiong N., Yao Y., Li X.Q., Wei S.Z. (2022). Dynamic recrystallization behavior of a Mo-2.0%ZrO_2_ alloy during hot deformation. Int. J. Refract. Met. Hard Mater..

[40] Gao P.F., Fu M.W., Zhan M., Lei Z.N., Li Y.X. (2020). Deformation behavior and microstructure evolution of titanium alloys with lamellar microstructure in hot working process: A review. J. Mater. Sci. Technol..

[41] Sander B., Raabe D. (2008). Texture inhomogeneity in a Ti-Nb-based beta-titanium alloy after warm rolling and recrystallization. Mater. Sci. Eng. A Struct. Mater. Prop. Microstruct. Process..

[42] Manohar P.A., Lim K., Rollett A.D., Lee Y. (2003). Computational exploration of microstructural evolution in a medium C-Mn steel and applications to rod mill. ISIJ Int..

[43] Lin Y.C., Chen X.M., Wen D.X., Chen M.S. (2014). A physically-based constitutive model for a typical nickel-based superalloy. Comput. Mater. Sci..

