# Supplementary material for: Dynamic Recrystallization Constitutive Model and Texture Evolution of Metastable β Titanium Alloy TB8 during Thermal Deformation
Source: Materials (Basel). 2024 Mar 29;17(7):1572. doi: 10.3390/ma17071572 (PMC11012458; doi:10.3390/ma17071572)
Supplement: Supplementary file 1 [file materials-17-01572-s001.zip › materials-2929100-supplementary.pdf]

Attachment

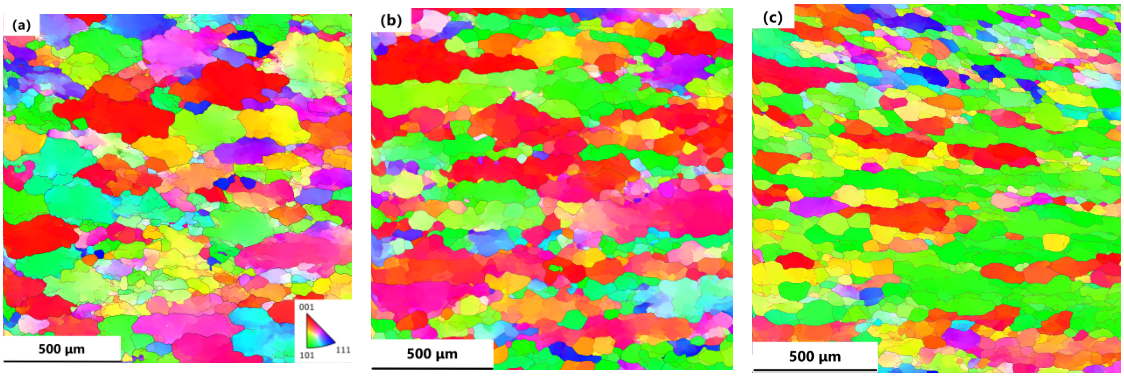

Figure S1 Inverse pole figure of TB8 titanium alloy under different deformation: (a)30%; (b)50%; (c)70%

Dynamic recrystallization constitutive model

In the process of thermal deformation, the flow stress σ can be expressed as a function of strain rate, strain ε, and deformation temperature T:

$$\sigma = f(\dot{\varepsilon}) f(\varepsilon) f(T) \tag{1}$$

Sellars believes that the relationship between flow stress σ and strain rate, strain ε and deformation temperature T can be expressed by hyperbolic sine function under high temperature thermal deformation.

$$\dot{\varepsilon} = A\left[\sinh\left(\alpha\sigma_p\right)\right]^n \exp\left(-Q_a / RT\right) \tag{2}$$

According to Galiyev, the relationship between flow stress σ and strain rate, strain ε and deformation temperature T at low strain rate can be expressed as:

$$\dot{\varepsilon} = A\sigma_P^n \exp\left(-Q_a / RT\right) \tag{3}$$

Mwembela believes that the relationship between flow stress σ and strain rate, strain ε and deformation temperature T at high strain rate can be expressed as:

$$\dot{\varepsilon} = A \exp\left(\beta\sigma_P\right) \exp\left(-Q_a / RT\right) \tag{4}$$

σ_P is the peak stress, Q_a is the deformation activation energy (J/mol) R=8.134J/ (mol·K), T is the mechanical temperature (K), α, β, n, A are the parameters of the material itself. Logarithms were taken on both sides of the above formula, and the results obtained by linear regression were shown in Table 1

Table S1 linear regression analysis values of n1, β and n

	n_1	β	n
780	4.375044	0.03089	2.41164
840	3.71953	0.03626	2.4988
900	3.51992	0.05097	2.84737
960	3.97281	0.07058	3.43154

average	3.896826	0.047175	2.797338

For Eq. (2), when the deformation rate is constant, Qa/Rn can be obtained by linear regression, and Q_a/Rn at multiple strain rates can be obtained by changing the strain rate. The average value of Q_a/Rn can be obtained by adding R and n into Q_a/Rn, as shown in Table 2, and Q_A =239147J/mol.

Table S2 Q_a/Rn linear regression analysis values

strain rate	0.1	0.01	0.001	average
Q_a/Rn	12695.71405	8684.08382	9468.51911	10282.77233

Zener and Hollomon proposed that in addition to the material itself, the stress-strain relationship is mainly affected by the deformation temperature T and strain rate, which can be expressed by the Zener-Hollomon parameter:

$$Z = \dot{\varepsilon} \exp\left(Q_a / RT\right) \tag{5}$$

The relationship between the peak strain ε_p and the Z parameter can be expressed as follows:

$$\varepsilon_p = K_p Z^{-P} \tag{6}$$

Where ε_p can be obtained from the stress-strain curve, the biased linear fit to ε_p and Z can be obtained as follows: K_p=1.730813, P=0.14929.

The relationship between the critical strain ε_c and the peak strain ε_p can be expressed as follows:

$$\varepsilon_c = \lambda \varepsilon_p \tag{7}$$

Where λ is a parameter of the material itself, most alloys are in the range of 0.67-0.89, for TB8 titanium alloy take 0.83 for calculation.

In this paper, we use the equation proposed by Laasraoui et al. based on the relationship between DRX and stress softening to obtain X_{drx}[1]:

$$X_{drx} = \frac{\sigma_s - \sigma}{\sigma_s - \sigma_{ss}} \tag{8}$$

The saturated strain σ_s and steady state strain σ_{ss} can be expressed in terms of hyperbolic sine functions applicable to all stress cases. The σ_s and σ_{ss} in terms of the Z-parameter can be expressed as:

$$\begin{cases} \sigma_s = 86.6446 \times \sinh^{-1}(n_s Z^{d_s}) \\ \sigma_{ss} = 86.6446 \times \sinh^{-1}(1.26 \times 10^{-3} Z^{0.30876}) \end{cases} \tag{9}$$

where n_s and d_s are parameters of the material itself, which can be obtained by quadratic fitting to the strain rate:

$$\begin{cases} d_s = 0.01376 \times \ln \dot{\varepsilon}^2 + 0.14417 \ln \dot{\varepsilon} + 0.60966 \\ n_s = -0.14396 \times 10^{-4} \times \ln \dot{\varepsilon}^2 - 0.00806 \ln \dot{\varepsilon} - 0.136 \end{cases} \quad (10)$$

According to JMAK kinetic theory[2], the kinetic model of dynamic recrystallization is expressed as follows:

$$X_{drx} = 1 - \exp\left[-K_d \left(\frac{\varepsilon - \varepsilon_c}{\varepsilon_p} \right)^{p_d} \right] \quad (11)$$

Where d_1, m_d are parameters of the material itself. Derivation of the above equation gives：

$$Ln\left[-Ln\left(1 - X_{drx}\right)\right] = Lnd_1 + m_d Ln\left(\frac{\varepsilon - \varepsilon_c}{\varepsilon_p} \right) \quad (12)$$

A linear regression analysis of the above equation yields K_d, P_d for different deformation conditions, and a biased linear fit to the Z parameter:

$$K_{\mathrm{d}} = 66.34577 Z^{-0.19488} \quad (13)$$

$$P_{\mathrm{d}} = 3 \times 10^{-4} Z^{-0.31595} \quad (14)$$

From the stress-strain diagram, it can be seen that the elastic phase of TB8 titanium alloy conforms to the shear Hooke's law can be expressed as:

The relationship between theyield stress and the Z parameter can be expressed as follows:

$$\sigma_0 = k_0 \cdot Z^{d_0} \quad (15)$$

Where σ_0 can be obtained from the stress-strain curve, the biased linear fit to εp and Z can be obtained as follows: k_0=0.33511, d_0=0.26082.

Based on the dislocation density theory, the evolution of dislocation density ρ with ε during the initial stage of thermal deformation, i.e., the hardening stage, can be expressed as[3]:

$$\frac{d_\rho}{d_\varepsilon} = U - \Omega \rho \quad (16)$$

Where ρ represents the dislocation density, U denotes the work-hardening coefficient, and Ω signifies the dynamic softening coefficient. The stress needed for the material to undergo high-temperature plastic deformation is principally determined by the proliferation of dislocations and inter-dislocation resistance. The relationship between flow stress and dislocation density can be expressed by the Taylor relationship as[3]:

$$\sigma = \alpha \mu b \sqrt{\rho} \quad (17)$$

where α represents the parameter of the material, μ is the shear modulus, and b is the Burgers vector. This can be obtained by bringing Eq. (15) into Eq. (14) and integrating:

$$\sigma_w = \left[\sigma_s^2 + \left(\sigma_0^2 - \sigma_s^2 \right) e^{-\omega \varepsilon} \right]^{0.5} \cdots\cdots \left(\varepsilon_q < \varepsilon \le \varepsilon_{\mathrm{c}} \right) \quad (18)$$

Where σ_0 represents the yield stress and ω denotes the dynamic softening amount. The experimentally obtained stress and strain values on the flow stress curve prior to reaching the critical strain can be chosen. These values can be substituted into the equation above to determine the value of ω under varying strain conditions. This can be obtained by plotting the lnω-lnZ relationship and fitting it to a line row:

$$\omega = 0.282995 \cdot Z^{0.1341} \tag{19}$$

When the critical strain is reached, DRX starts to appear in TB8 titanium alloy, and the rheological stress model for the DRX stage can be obtained according to Eqs. (11) and (20):

$$\sigma_{\mathrm{d}} = \sigma_w - \left(\sigma_s - \sigma_{ss}\right) X_{drx} \cdots\cdots \left(\varepsilon_c < \varepsilon\right) \tag{20}$$

The relationship between the dynamically recrystallized grain size D_{drx} and the Z parameter can be expressed by the following equation:

$$D_{drx} = CZ^h \tag{21}$$

Where C and h are intrinsic parameters of the material. By taking logarithms on both sides of the equation mentioned above, linear regression shows that h= -0.3601 and C=1.45*10^4.

References

1. El Wahabi M, Cabrera J M, Prado J M. Hot working of two AISI 304 steels: a comparative study. Materials Science and Engineering a-Structural Materials Properties Microstructure and Processing. **2003**, 343, 116-25.
2. Manohar P A, Lim K, Rollett A D, Lee Y. Computational exploration of microstructural evolution in a medium C-Mn steel and applications to rod mill. Isij International. **2003**, 43, 1421-30.
3. Lin Y C, Chen X M, Wen D X, Chen M S. A physically-based constitutive model for a typical nickel-based superalloy. Computational Materials Science. **2014**, 83, 282-9.
